# Long-term effects of a single adult methamphetamine challenge: Minor impact on dopamine fibre density in limbic brain areas of gerbils

**DOI:** 10.1186/1744-9081-2-12

**Published:** 2006-03-28

**Authors:** Susanne Brummelte, Thorsten Grund, Andrea Czok, Gertraud Teuchert-Noodt, Jörg Neddens

**Affiliations:** 1 Department of Neuroanatomy, Faculty of Biology, University of Bielefeld, Universitätsstr. 25, D-33615 Bielefeld, Germany; 2 National Institutes of Health, NICHD, Section on Molecular Neurobiology, Bldg. 35, Rm. 2C-1004, Bethesda, MD 20892-3714, USA

## Abstract

**Background:**

The aim of the study was to test long-term effects of (+)-methamphetamine (MA) on the dopamine (DA) innervation in limbo-cortical regions of adult gerbils, in order to understand better the repair and neuroplasticity in disturbed limbic networks.

**Methods:**

Male gerbils received a single high dose of either MA (25 mg/kg i.p.) or saline on postnatal day 180. On postnatal day 340 the density of immunoreactive DA fibres and calbindin and parvalbumin cells was quantified in the right hemisphere.

**Results:**

No effects were found in the prefrontal cortex, olfactory tubercle and amygdala, whereas the pharmacological impact induced a slight but significant DA hyperinnervation in the nucleus accumbens. The cell densities of calbindin (CB) and parvalbumin (PV) positive neurons were additionally tested in the nucleus accumbens, but no significant effects were found. The present results contrast with the previously published long-term effects of early postnatal MA treatment that lead to a restraint of the maturation of DA fibres in the nucleus accumbens and prefrontal cortex and a concomitant overshoot innervation in the amygdala.

**Conclusion:**

We conclude that the morphogenetic properties of MA change during maturation and aging of gerbils, which may be due to physiological alterations of maturing vs. mature DA neurons innervating subcortical and cortical limbic areas. Our findings, together with results from other long-term studies, suggest that immature limbic structures are more vulnerable to persistent effects of a single MA intoxication; this might be relevant for the assessment of drug experience in adults vs. adolescents, and drug prevention programs.

## Background

Methamphetamine (MA) is a common illicit drug, which abuse is currently reaching epidemic proportions. According to the 2002 SAMHSA National Household Survey on Drug Abuse, 12.4 million Americans age 12 and older had tried methamphetamine at least once in their lifetimes (5.3 percent of the population). This increasing number is especially alarming since it has been extensively shown that MA exerts acute neurotoxic effects on the monoaminergic transmitter systems, and thus leads to characteristic cognitive impairments like deficits in memory and learning, psychomotor speed and information processing [1]. It is especially affecting the dopamine (DA) neurons, leading to dramatic loss of fibres and other DAergic structures in certain brain areas within a few days [2,3], even after a single exposure [4].

Some evidence exists that monoaminergic fibres are able to recover to some extend from this damage during long time course [5-9]. Moreover, even reactive overshoot was found for serotonergic fibres in several limbic areas of the brain, including left entorhinal cortex [10] and the septal pole of the hippocampal dentate gyrus [11]. For DA fibres, an early MA treatment produces hyperinnervation in amygdaloid nuclei and ventral entorhinal cortex [12] and a restraint of the maturation in prefrontal cortex [13,14]. This lab has further shown that the single early MA intoxication produces a loss of DA fibres and concomitant hyperinnervation of serotonin fibres in the nucleus accumbens (NAC) [15].

Taken together, our recent studies indicate severe changes in the maturation of the limbo-cortical network following an early single MA challenge. However, a primary study has already shown that the neuroplasticity that follows MA treatment might relate on the age of the animals [4]. Since the functional maturation and aging of the brain is based on various structural and physiological changes, the present study was carried out to question whether the remodelling of neural networks that is induced by the neurotoxic effects of MA may alter during the lifespan of gerbils. For that purpose, 6 months old adult gerbils received a single high dose of MA. At the age of 12 months the DA innervation was examined in prefrontal cortex, olfactory tubercle, NAC, and amygdala to check for long-time effects on the fibre density.

## Methods

All experimental procedures were approved by the appropriate committee for animal care in accordance with the guidelines of the European Communities Council Directive. Breeding gerbils *(Meriones unguiculatus) *were obtained from Harlan Winkelmann (Borchen, Germany). From offspring, a total of 18 males (weight 66–91 g; age 331–348 days) were used in this study. Young animals were weaned at postnatal day 30 and subsequently separated in standard cages (Macrolon^® ^type 4) without any content except of sawdust. All animals had free access to food and water and were kept on natural day/night cycles. On postnatal day 180, a total of 9 gerbils received a single systemic injection of (+)-methamphetamine hydrochloride (Sigma, M 8750; 25 mg/kg, i.p.). The other 9 animals were sham-treated by an i.p. injection of saline. This dose was chosen due to our former experiences, which have shown that juvenile gerbils can tolerate higher doses (50 mg/kg) than older ones. Notably, the rate of mortality is similar at both ages receiving the different doses (unpublished data), indicating physiological changes during the postnatal maturation of the brain.

The methods used for sectioning and DA immunohistochemistry have been published recently [15]. For the immunohistochemistry of calbindin and parvalbumin cells, 50 μm thick vibratome sections were taken from the same animals (perfused with 100 ml 0.1 M sodium cacodylate pH 6.2, followed by 750 ml 5% glutaraldehyde in 0.1 M sodium cacodylate pH 7.6) and treated as follows: Every third section was collected in 0.05 M Tris-HCL buffered saline [TBS (pH 7.5)] at 4°C; rinsed 3 × 10 min in TBS; incubated 10 min with 1% H2O2 in TBS; rinsed again 3 × 10 min in TBS; blocked in 10% normal goat serum and 0.4% Triton X-100 (Sigma) for 30 min; incubated with the primary antibody (1:3,000 mouse anti-calbindin, Sigma; 1:2,000 mouse anti-parvalbumin, Sigma) in 1% normal goat serum and 0.4% Triton X-100 for 18 h; rinsed 3 × 10 min in TBS; incubated for 30 min in biotinylated goat-anti-mouse antibody (Sigma) diluted 1:20 with 1% normal goat serum; rinsed 3 × 10 min in TBS; incubated with ExtraAvidin-Peroxidase (Sigma) diluted 1:20 for 30 min; rinsed 3 × 10 min in TBS; stained in 0.05% 3.3-diaminobenzidine (Sigma) with 0.01% H2O2 for 4 min. Finally, the sections were rinsed 5 × 10 min in TBS, mounted on glass slides, dried overnight, dehydrated with ethanol, cleared with xylene and cover slipped with DePeX (Serva, Heidelberg, Germany). To avoid deviations due to probably lateralised innervation densities of DA or calcium-binding proteins only right hemispheres were used for quantification.

**Figure 1:**
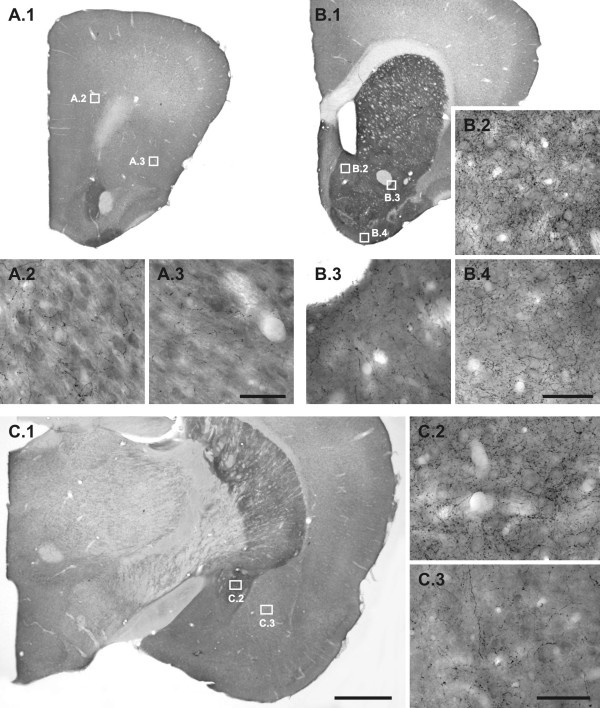
**Dopamine immunoreactive fibres in each of the quantified regions**. Representative photomicrographs, taken from a saline control, of dopamine (DA) immunoreactive fibres of each of the quantified regions. A.1: Prefrontal cortex; A.2: Layer VI of the prelimbic area; A.3: Layer IV of the lateral orbital and agranular insular areas. B.1: Nucleus accumbens (NAC); B.2: Medial shell of NAC; B.3: Lateral core of NAC; B.4: Olfactory tubercle. C.1: Amygdala (AMY); C.2: Central nucleus of AMY; C.3: Basolateral nucleus of AMY. Note the differential innervation pattern and density of DA fibres in the respective regions. Scale bars: 1000 μm (A.1, B.1, C.1); 50 μm (A.2-3, B.2-4, C.2-3).

For quantification of fibre and cell densities, brain sections were chosen in areas of interest by means of anatomical characteristics according to brain atlases of the rat [16] and the mouse [17]. The identification of the brain region follows the nomenclature of the atlas of the rat. The average number of analysed sections was 18 per animal for DA, with a range of 4 up to 6 sections in single regions. In the defined region of each section (cf. Fig. 1) all detectable fibre fragments and cells were visualised in standard test fields (2,080 × 1,544 pixel; 0.22 mm^2^) using a bright field microscope (BX61, Olympus, Hamburg, Germany) and a digital camera for microscopy (ColorView II, SIS, Münster, Germany) at 200-fold magnification. Cells and fibres were quantified by software for image analysis (KS300, Jenoptik, Jena, Germany). Immunoreactive DA fibres of different diameter were standardised to identical thickness and visualised using a combination of Gauss filter and Gerig operator that depicts differences of grey values of adjacent pixels and transforms the result into binary images. The DA fibre density was computed as a percentage of the evaluated test area. Calbindin and parvalbumin positive cells were detected by use of a threshold to the grey value, followed by automatic sorting of adequate shape and minimal size (250 pixels) of the structures. Remaining structures were classified as cells, the size of the structures (cell area) being measured cumulatively and the according cell density calculated by proportion of cell number per test field area. Calbindin-positive cells are located almost exclusively in the core region of the NAC and were measured only in this part of the NAC, whereas medium-sized PV-positive cells are specific to the shell and were counted only in this area. All analyses were done by a person blind to the pharmacological treatment of individual animals.

**Figure 3:**
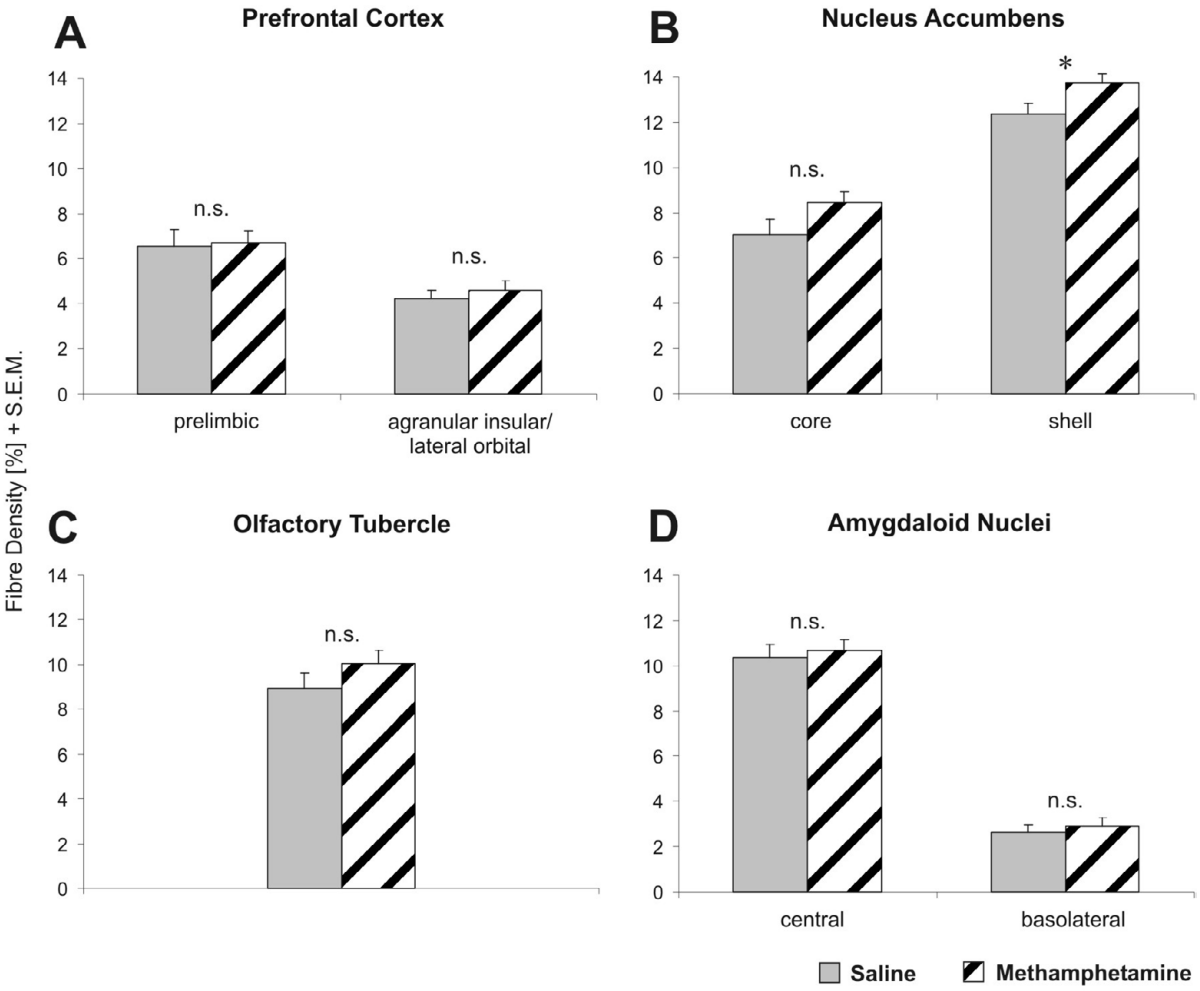
**Dopamine innervation density in four regions of the gerbil brain**. Dopamine (DA) innervation density ± S.E.M. is presented in four regions of the gerbil brain, namely agranular insular and lateral orbital as well as prelimbic areas of the prefrontal cortex, the olfactory tubercle, core and shell areas of the nucleus accumbens (NAC), and the central and basolateral nuclei of the amygdala complex. Methamphetamine treatment generally tends to increase the DA innervation. However, a significant region-specific change in response to a single adult methamphetamine treatment exclusively occurs in the shell of the NAC (+11%; *p *= 0.0332). The difference in the core appears somewhat more pronounced but is not significant due to higher variance (+21%; *p *= 0.1011). Student's t-Test, significance value: * *p *< 0.05. Following methamphetamine treatment, ANOVA detected a significant overall increase of DA innervation in core and shell of the NAC (F(1,16) = 4.7316; *p *= 0.0472).

The measurements were computed as arithmetic means by-case and by-group ± S.E.M. of the respective regions (Fig. 3). Statistical analysis revealed regional effects of MA treatment by the use of Student's t-test. General alterations in the NAC were additionally investigated by use of 2-way analysis of variance (ANOVA), which checked for area-specific and group-specific effects [18]. Data analysis was computed with Statistica 6 (StatSoft, Tulsa, USA). The levels of significance were set at * *p *< 0.05, ** *p *< 0.01, and *** *p *< 0.001.

## Results

The innervation pattern of DA immunoreactive fibres in gerbils is generally in line with the results of rats. The innervation pattern and density of DA immunoreactive fibres in the gerbil forebrain are region-specific (Figs. 1 and 3). Representative photographs of the differential DA innervation densities and patterns of the four regions that were subsequently studied in more detail are provided in Fig. 1, taken from a male gerbil of the saline group.

Quantitative DA data were obtained from a total of 327 sections that derived from 18 gerbils of two experimental groups (Saline n = 9, MA n = 9). The adult single systemic MA challenge induces no general alteration of DA innervation pattern in the investigated regions of the gerbil brain (Fig. 3). The overall DA fibre density in the NAC is selectively increased by MA [(ANOVA, F(1,16) = 4.7316, p = 0.0472) please note that ANOVA included comparison of 8 vs. 8 animals only, because some NAC sections were damaged in one animal of each experimental group]. However, the significant increase (+11%) is limited to the shell (Student's t-test, *p *= 0.0332), whereas alteration in the core misses statistical significance (Student's t-test, *p *= 0.1011). No change in DA fibre density was found in the prefrontal cortex, olfactory tubercle and amygdala.

**Figure 2:**
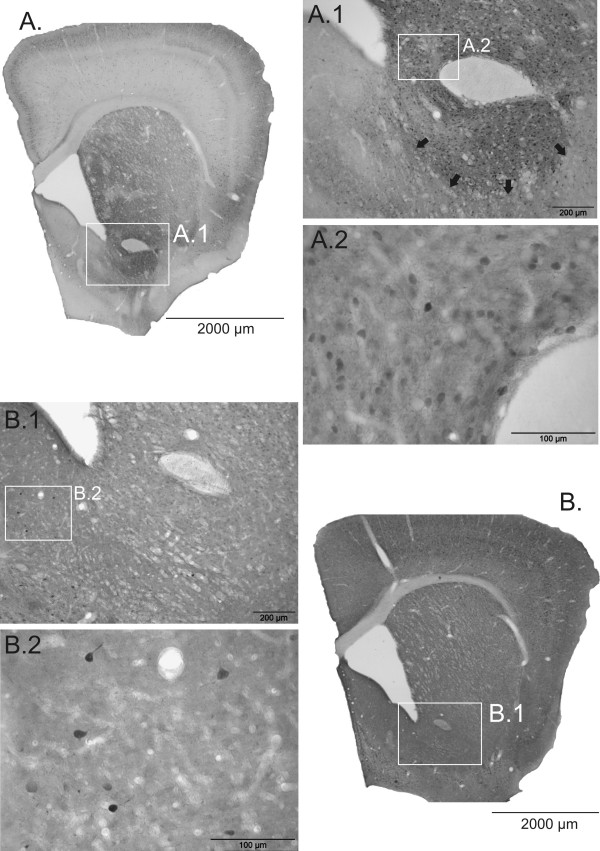
**Photomicrographs of Calbindin and Parvalbumin immunoreactive neurons in the nucleus accumbens**. Overview (A) and higher magnifications (A1, A2) of the Calbindin innervation of the NAC. The majority of CB+ cells is located in the core, which border to the shell is detectable (black arrows). PV+ cells are almost exclusively located in the shell (B1, B2), however, the overall density is much lower compared to CB+ cells. Scale bars: 2000 μm (A, B); 200 μm (A1, B1); 100 μm (A2, B2).

**Figure 4:**
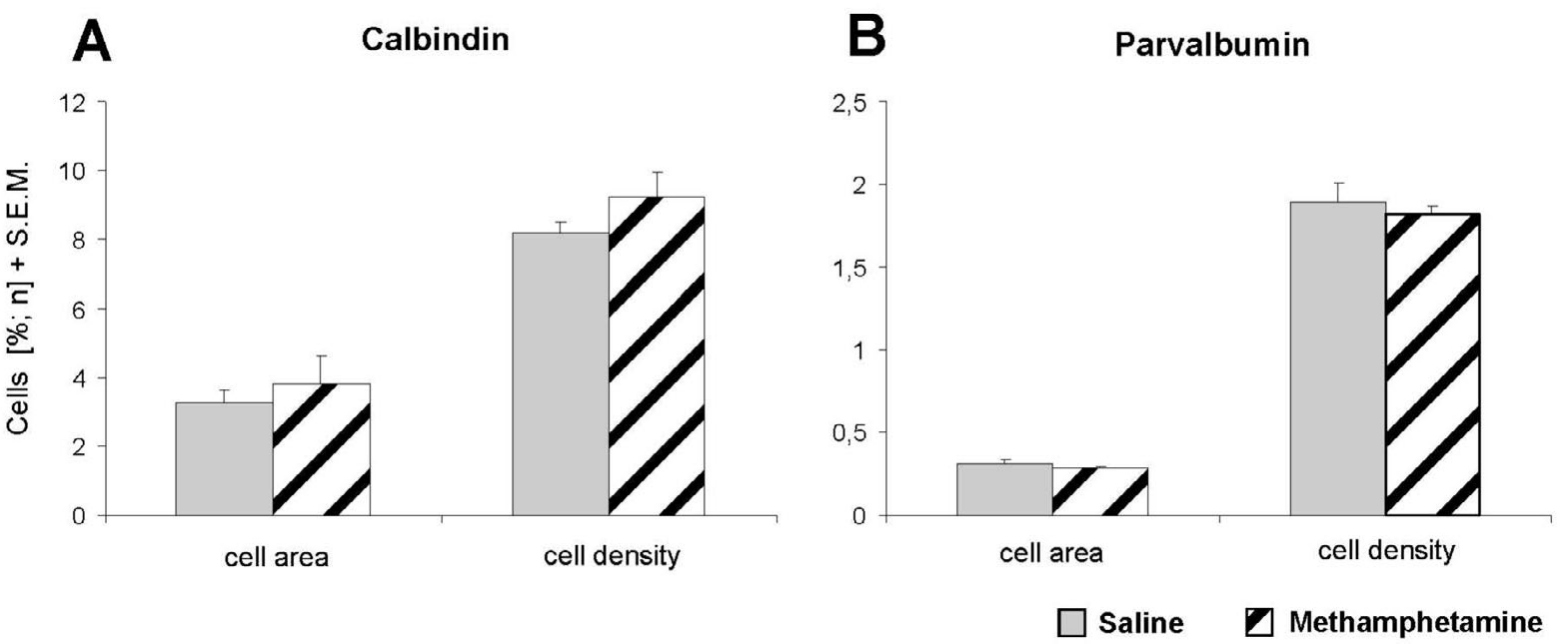
**Calbindin and parvalbumin cell densities and cell areas in the nucleus accumbens**. Calbindin (CB) and parvalbumin (PV) cell densities and cell areas ± S.E.M. are presented for the nucleus accumbens (NAC). PV-positive cells and CB-positive cells are predominantly located in the shell and in the core of the NAC, respectively, where they were quantified. No statistically significant effect of a single adult methamphetamine challenge could be detected for either number (cell density) or cumulative size (cell area) of both PV and CB cells. Generally, the number of CB cells is considerably higher and their average size is doubled compared to PV cells.

To determine other potential alterations within the NAC, CB- and PV- positive structures were additionally investigated in this area. The distribution of CB- and PV-positive subpopulations in the gerbil generally resembles the distribution in the NAC of rats and primates (Fig. 2) [19-21]. A single adult MA intoxication caused no significant alteration in the cell density of either CB- positive neurons or PV-positive neurons in the NAC. Neither was there a difference in the cell areas (Fig. 4).

## Discussion

According to our results, a single adult MA challenge induces minor long-term changes of the DA innervation in the NAC, whereas other regions of the limbocortical circuitry are apparently unaffected. These results contrast with previously published data on the long-term effects of early postnatal MA treatment that demonstrated a restraint maturation of DAergic fibres in the NAC and the prefrontal cortex [14,22] and a concomitant overshoot innervation in the amygdala [12]. Table 1 provides a comparison between MA effects on DA innervation in juvenile and adult gerbils.

**Table 1:** Comparison of age-related long-term effects of a single methamphetamine intoxication on the dopamine innervation in limbocortical areas of the gerbil brain.

		Adult (current study) MA 1 × 25 mg/kg i.p., p180	Juvenile [Reference] MA 1 × 50 mg/kg i.p., p14
Prefrontal cortex	Medial	↔ n.s.	↓ – 38% ** [14]
	Orbital	↔ n.s.	↓ – 50% ** [14]
Nucleus accumbens	Core	↔ n.s.	↓ – 28% * [15]
	Shell	↑ +11% *	↔ n.s. [15]
Amygdala	Basolateral	↔ n.s.	↑ +18% ** [12]
	Central	↔ n.s.	↔ n.s. [12]
Olfactory Tubercle		↔ n.s	No data

Significance values: * *p *< 0.05, ** *p *< 0.01.

### Postnatal development and vulnerability to methamphetamine

The age-related and region-specific alterations that are triggered by a single MA-treatment of gerbils might reveal the complexity of MA neurotoxicity. It has to be pointed out that the different doses that were administered to juvenile (50 mg/kg) [10-15,22] vs. adult (25 mg/kg, current study) animals may be even more adequate for comparing age-related effects than using the same dose in both ages, because a lethal dose of MA is approximately also twice as high in juvenile gerbils compared to adults (unpublished data), indicating physiological changes during postnatal maturation of the brain. The reasons for the age-dependent differences in vulnerability to MA in gerbils are currently not clear. However, it appears reasonable to assume that this is related to physiological alterations in maturing vs. mature monoaminergic neurons. Generally, the high amount of MA that is required to induce such effects might be specific to gerbils, probably due to species-specific metabolic enzymes.

Although the exact molecular mechanism of MA neurotoxicity is still not completely understood, it is clear that developmental alteration must play an important role in mediating the MA-induced effects [23]. This is demonstrated by the finding that the application of MA results in higher mortality and stronger reactions of adult gerbils compared to juveniles or adolescents [4,24-29], which may be understandable by reports that, in rats, higher MA concentrations occur in the brains of 90 days old versus 40 days old animals after receiving the same dose [25,29]. However, Kokoshka and colleagues published some intriguing results which on the one hand confirmed previous studies concerning the lack of medium-term (7 days) deficits in the DA systems after MA treatment in adolescent rats, but on the other hand showed that there were acute short-term (1 hour) consequences in adolescent (40 days) and adult (90 days) rats [25]. Further, MA-induced behavioural sensitization, which is a prominent feature of MA administration [30,31], seems to be age-dependent [32]. It does not occur within a crucial postnatal period, which in turn seems to correspond to the time of presynaptic DA autoreceptor formation in the brain [33].

Several parameters of the DA system underlie developmental changes, e.g. DAT expression [34,35], expression of DA receptors and DA concentration [36], and activity of the vesicular monoamine transporter-2 (VMAT-2) [29]. The mechanism underlying the modifications seen in adult animals after MA challenge is therefore thought to vary from the one mediating the neurotoxic effect in juvenile animals. The ability of a single early MA challenge to selectively induce a restraint of the maturation of DA fibres in the prefrontal cortex and the NAC [14,22,37] as well as a concomitant excessive maturation in several amygdaloid nuclei and the entorhinal cortex [12] might be due to a special vulnerability of immature fibre systems [38]. As DA transmission in the NAC seems to play a critical role in an input selection mechanism that regulates the influence of certain inputs over neural activity [39], the reactive changes that occur within local circuits following the MA challenge might cause a new and different innervation pattern of these fibres and thus a neuroanatomical restructuring [40,41]. The severe impairment of the brain architecture induced by a single early MA treatment clearly demonstrates that despite the apparent higher resistance of younger animals, MA is indeed a potent drug capable of inducing extensive structural alterations in the juvenile brain that persist into adulthood.

### Effects of methamphetamine on different neurotransmitters and brain regions

It was reported that the mechanism of MA neurotoxicity includes the formation of reactive oxygen [42-44] and nitrogen [42,45,46] species, which damage monoaminergic neurons. However, several other factors may also contribute in mediating the neurotoxic effect of MA, leading to region-specific and neuron-specific differences in vulnerability. Fumagalli and colleagues have shown that rats lacking the dopamine transporter (DAT) are protected against the MA-related neurotoxicity in the striatum [47]. Interestingly, impairment in the function of VMAT-2, which accumulates cytoplasmic DA into synaptic vesicles as seen in mice heterozygous for this transporter, increases the MA neurotoxicity [48]. It has also been demonstrated that the blockage of either DA D1 or D2 receptors prevents the damage of repeated doses of MA to striate DA terminals [49] and that there are regional differences in sensitivity of these terminals to the MA [4,50]. It seems likely that DAT and DA receptors may be factors limiting the severity of neurotoxic effects of MA, presumably by influencing the concentration and distribution of DA.

MA-induced alterations have also been found in other neuronal elements like 5-HT fibres [10,15,51,52], GABAergic neurons [53], and the morphology of cortical pyramidal cells [54]. Our animal model has also revealed that glutamatergic projection fibres from the mediodorsal prefrontal cortex to several other cortical areas are significantly reduced after an early single MA intoxication [55]. Interestingly, in the present study the shell of the NAC is the only area that reacts to a single adult MA challenge, and it is also almost the only area we have studied lacking any effect of the DA fibre density in response to a single postnatal drug treatment [12,14,15] (cf. Table 1). In contrast, the core region of the NAC exhibits a strong decline in DA fibre density after an early single MA administration [15]. This is in line with results from Broening and colleagues, who, after repeated MA treatment of rats, found an almost completely loss of tyrosine hydroxylase immunoreactivity in the core while the shell was almost spared [56]. In addition, most drugs increase extracellular DA levels preferentially in the shell region of the NAC [57], which coincides with differences in DA baseline levels [57,58], and different time-course of the maturation of the DA innervation in the core and shell areas [59]. We may conclude that the DA fibre systems of the brain are far from being homogeneous; thus, statements on the general effects of MA intoxication on DA fibres are misleading.

### Regeneration and reorganisation of neural networks: implications for psychiatric diseases

It has been shown before that DA fibres can be rebuilt within 24 weeks after a lesion of the NAC with 6-OHDA [60]. Furthermore, Finkelstein and colleagues could show that a lesion to the substantia nigra causes sprouting of DA fibres in the striatum of rats [61]. Thus, the increased fibre density we have found in the NAC might probably be caused by a specific regeneration rather than a reorganisation of fibres.

Several studies in humans and rodents have shown that the effects of MA are to a large extend reversible, although this process might last many years and may strongly depend on the severity and duration of the drug abuse [1]. We have shown that a single administration of MA on postnatal day 90 leads to a transient increase of the dendritic spine density of prefrontal pyramidal neurons, which regain an almost normal level within 30 days post-treatment [62]. In the present study, we apparently provided sufficient time for the impaired system to recover from MA intoxication and to eventually regain normal DA fibre densities. Our model using only a single administration of MA may therefore be more useful to mimic the effects on first time users rather than on chronically abusers of the drug. In addition, it has been shown that intermittent treatment with MA can lead to the development of tolerance to its neurotoxic effects [63-65]. Thus the paradigm of repeated administration of MA as used in the majority of studies might either conceal or modify the deleterious effects of the psychostimulant. In fact, some studies have shown opposed or stronger effects of single versus chronic administration of MA [66,67].

Chronic MA use is known to cause psychotic symptoms that mimic that of schizophrenia [68-70]. Further, we could recently show that an early single intoxication leads to a 'dysconnection' of prefrontal efferents [55], thus providing an anatomical correlate of schizophrenia [71,72]. This is consistent with results from Chen and colleagues who revealed an association between earlier and larger use of MA with higher risk of psychosis in humans [68].

## Conclusion

The results of this study show that even a single application of MA to adult gerbils may induce long-term physical alterations in limbic brain areas, although the effects are not as severe as seen after an early drug challenge. The increased DAergic fibre density in the NAC indicates reactive over-sprouting, which possibly is a response to altered network requirements after MA treatment. It remains to be investigated whether other brain areas would reveal short-term modifications, which might be concealed by recovery in this approach. While it cannot be excluded that the different effects in adult vs. juvenile gerbils are, at least in part, due to the different doses of MA, several other studies also indicate changes in MA effects that depend on the age of rats and mice. We thus may conclude that, in rodents, MA not only acts age-dependent, but also highly region-specific. We have sufficient evidence to suggest that early contact with this psychotropic substance during childhood might increase the risk of persistent severe structural changes of the brain architecture and may result in long-term cognitive and psychiatric disturbances.

## Competing interests

The author(s) declare that they have no competing interests.

## Authors' contributions

SB participated in the interpretation of the data and drafted the manuscript.

TG contributed to the benchwork, analysis and interpretation of the data.

AC contributed to the acquisition and interpretation of data.

GT contributed to the design of the study and the critical reviewing of the manuscript.

JN handled the animals, contributed to the benchwork, analysis and interpretation of the data, participated in the design of the study, and the drafting and revision of the manuscript.
